# Diseases Caused by Parasites with Invertebrate Hosts in China: Burden and Trends of Leishmaniasis and Schistosomiasis

**DOI:** 10.3390/pathogens15030340

**Published:** 2026-03-23

**Authors:** Cun-Chen Wang, Shu-Jing Wang, Rui Han, Gui-Zhi Xu, Hai-Ting Zhang, Xin-Xue Zhu, Qi-Long Wu, Yi-Xue Zhao, Yu-Jie Zhou, Zhen-Zhong Feng, Miao Liu, Sheng-Qun Deng

**Affiliations:** 1 Department of Pathology, The Second Affiliated Hospital of Anhui Medical University, Hefei 230601, China; 2 Department of Pathogen Biology, Anhui Province Key Laboratory of Zoonoses, The Provincial Key Laboratory of Zoonoses of High Institutions in Anhui, School of Basic Medical Sciences, Anhui Medical University, Hefei 230032, China; 3 Clinical College of Anhui Medical University, Hefei 230031, China

**Keywords:** leishmaniasis, schistosomiasis, China, disease burden, GBD2021

## Abstract

Parasitic diseases involving invertebrate hosts, notably leishmaniasis (transmitted by sandflies) and schistosomiasis (transmitted via aquatic snails), remain public health concerns in China. Based on the Global Burden of Disease 2021 data, the age-standardized prevalence, mortality, and disability-adjusted life years (DALY) rates for both diseases declined significantly from 1990 to 2021. Men consistently experienced a higher burden than women. The age distribution of disease burden differed between the two conditions. Projections to 2036 suggest a continued decrease in schistosomiasis burden and in leishmaniasis mortality and DALYs, but a potential slight rise in leishmaniasis prevalence. Sustained control efforts have been effective, yet challenges persist due to demographic disparities, climate-related invertebrate host/intermediate host expansion, and imported cases. Future strategies require enhanced surveillance, targeted interventions, and multi-sectoral collaboration to advance toward elimination.

## 1. Introduction

Diseases caused by parasites with invertebrate hosts (PDIHs) refer to a category of illnesses caused by parasites that depend on specific invertebrate animals for their life cycle and transmission. This broad category includes classical vector-borne diseases transmitted by biting arthropods (e.g., mosquitoes, sandflies) as well as diseases like schistosomiasis, which utilizes aquatic snails as intermediate hosts for water-based transmission [1]. Their prevalence is influenced by natural, economic, and social factors, posing a significant threat to global public health. Although PDIHs typically do not present with acute onset, their widespread occurrence can impair the health and productivity of both animals and humans, posing a significant threat to global public health [2]. Major PDIHs include malaria, leishmaniasis, schistosomiasis, African trypanosomiasis, American trypanosomiasis, and others [3]. China was once among the countries most severely affected by parasitic diseases, with malaria, schistosomiasis, and filariasis historically endangering public health [4,5]. Since the founding of the People’s Republic of China, the government has prioritized the prevention and control of parasitic diseases, achieving effective management of PDIHs overall. For example, China received certification from the World Health Organization (WHO) for eliminating lymphatic filariasis in 2007 and malaria in 2021 [6,7]. However, certain PDIHs, such as leishmaniasis (a classic vector-borne disease) and schistosomiasis (a water-based disease with an invertebrate intermediate host), still pose transmission risks [8,9,10]. Leishmaniasis is caused by *Leishmania* parasites and transmitted through the bite of infected female sandflies, with dogs or rodents such as rats serving as reservoir hosts [11]. The main species of *Leishmania* in China are *Leishmania donovani* and *Leishmania infantum*, which primarily cause visceral leishmaniasis [12,13,14,15]. Among its clinical forms, visceral leishmaniasis is the most severe manifestation [16]. In China, the predominant form is visceral leishmaniasis, which includes three epidemiological subtypes: anthroponotic visceral leishmaniasis (AVL), mountainous-type zoonotic visceral leishmaniasis (MT-ZVL), and desert-type zoonotic visceral leishmaniasis (DT-ZVL) [17]. Currently, MT-ZVL has become the predominant form, with *Phlebotomus chinensis* as its primary vector. This shift is driven by the ecological suitability for sandfly proliferation, the role of domestic and stray dogs as reservoir hosts, and socioeconomic factors such as rural aging, vacant housing, and inadequate public awareness and vector control [18,19]. Case distribution shows a shifting trend toward central provinces such as Shanxi, Shaanxi, and Gansu [18,19,20,21]. Schistosomiasis, caused by parasites of the genus *Schistosoma*, is primarily transmitted through skin contact with freshwater contaminated by infectious cercariae released by aquatic snail intermediate hosts. It is endemic in Asia, Africa, and Latin America [22]. In China, the predominant species is *Schistosoma japonicum*, with *Oncomelania hupensis* snails serving as intermediate hosts [23]. Over the past seven decades, China has made remarkable progress in schistosomiasis control. Current challenges include snail control, persistent transmission through animal reservoirs, and risks of imported cases [24,25].

In summary, although PDIHs in China have been effectively controlled, the distinct and persistent challenges posed by leishmaniasis and schistosomiasis highlight the necessity for a systematic analysis of the evolution of their disease burden. Therefore, using data from the Global Burden of Disease (GBD) 2021, we analyzed the disease burden and temporal trends of two major PDIHs (leishmaniasis and schistosomiasis) in China from 1990 to 2021, assessed their distribution disparities among populations, and projected the disease burden for the next 15 years. The findings are expected to provide a more nuanced understanding of the current challenges and offer key evidence for refining prevention and control policies, with the ultimate goal of mitigating the residual threats of these diseases and accelerating their elimination process.

## 2. Materials and Methods

### 2.1. Data Source and Extraction

The GBD 2021 study provides burden estimates for 371 diseases and injuries across 204 countries and territories from 1990 to 2021. These data are stratified by country and territory, year, sex, and age group [26]. Data related to parasitic diseases can be accessed via the Global Health Data Exchange (GHDx) Results Tool (available at https://gbd2021.healthdata.org/gbd-results/, accessed on 10 June 2025), which users can access directly through a web browser on its official website. We employed disability-adjusted life year (DALY) to quantify the disease burden. DALY is a core indicator used to measure the total disease burden in a population. It integrates the years of life lost due to premature death (YLL) with the years of healthy life lost due to disability (YLD) into a standardized metric. Each DALY represents the loss of one year of healthy life [10,27].

The data on prevalence, mortality, and DALYs for schistosomiasis and leishmaniasis were extracted from GBD 2021 using the following search parameters: Measure [Prevalence, Deaths, Disability-Adjusted Life Years (DALYs)], Metric (Number, Rate), Cause (Schistosomiasis, Leishmaniasis), Location (China), Age (All ages, Age-standardized, <5 years, 5–9 years, 5–14 years, 10–14 years, 15–19 years, 15–49 years, 20–24 years, 25–29 years, 30–34 years, 35–39 years, 40–44 years, 45–49 years, 50–54 years, 50–74 years, 55–59 years, 60–64 years, 65–69 years, 70–74 years, 75+ years, 75–79 years, 80–84 years, 85–89 years, 90–94 years, 95+ years), Sex (Both, Male, Female), Year (1990–2021, and each year from 1990 to 2021).

### 2.2. Definition of Diseases Caused by Parasites with Invertebrate Hosts

In China, following the successful elimination of malaria and lymphatic filariasis, leishmaniasis and schistosomiasis have emerged as the primary PDIH that still pose a risk of local transmission and entail a relatively substantial disease burden [8,10,21,28]. This study selects leishmaniasis and schistosomiasis as representative PDIHs in China for analysis, based mainly on the following considerations. First, after the elimination of malaria and lymphatic filariasis, these two diseases represent the major parasitic diseases in China that still carry a risk of indigenous transmission and a considerable disease burden, making them key targets for current prevention and control efforts. Second, the GBD 2021 provides systematic, continuous, and comparable data for both diseases, enabling long-term trend analysis and forecasting. Additionally, their current epidemiological status, stage of control, and existing challenges—such as the impact of climate change and the risk of imported cases—offer valuable insights for formulating sustainable control strategies for these parasitic diseases in China in the coming phase.

### 2.3. Statistical Analysis

Data analysis was performed using a linear regression model, incorporating natural logarithmic transformation. The model assumed a linear relationship between the natural log-transformed age-standardized rate (ASR, i.e., Y) and the calendar year (X), incorporating a random deviation (ε). The specific equation is Y = α + βX + ε. The β coefficient represents the direction and magnitude of the ASR change. To evaluate trends in age-standardized prevalence rate (ASPR), age-standardized mortality rate (ASMR), and age-standardized DALY rates (ASDR) for leishmaniasis and schistosomiasis in China (1990–2021), the estimated annual percentage change (EAPC) and its 95% confidence interval (CI) were calculated. The calculation formula is EAPC = 100 × (exp(β) − 1), with its 95% CI derived from the linear regression model. Trends were judged according to the following criteria: if the EAPC was positive and the lower limit of its 95% CI was also positive, it indicated an upward trend in ASR; if the EAPC was negative and the upper limit of its 95% CI was also negative, it indicated a downward trend; otherwise, the trend was considered stable [29,30].

Furthermore, the projection methodology employed an autoregressive integrated moving average (ARIMA) model to estimate China’s burden of leishmaniasis and schistosomiasis for 2022–2036. The ARIMA (p,d,q) model is a statistical method widely used for time series analysis and forecasting, where p denotes the order of the autoregressive term, d denotes the degree of differencing required to make the series stationary, and q denotes the order of the moving-average term [31,32]. The optimal ARIMA model was selected based on the Akaike Information Criterion (AIC) and the Bayesian Information Criterion (BIC), and its residuals were confirmed to follow an independent normal distribution using the Ljung–Box test. Based on historical data, the model predicted the ASPR, ASMR, and ASDR for leishmaniasis and schistosomiasis in China from 2022 to 2036. To evaluate the predictive performance of the ARIMA model, we employed a rolling forecast validation method based on annual data from 1990 to 2021. Starting from 2007, for each target year (t), an ARIMA model was constructed using actual values from 1990 to (t − 1) to forecast the value for year (t). This process was repeated until forecasted values for all years from 2007 to 2021 were obtained. The forecasting accuracy was quantified by comparing the predicted values with the actual values and calculating the mean absolute percentage error (MAPE). A MAPE value of less than 10% indicates a good level of forecast accuracy, with smaller values representing better predictive performance of the model. All statistical analyses were performed using the R program (version 4.4.2).

## 3. Results

### 3.1. The Prevalence Burden of Leishmaniasis and Schistosomiasis in China

**Figure 1:**
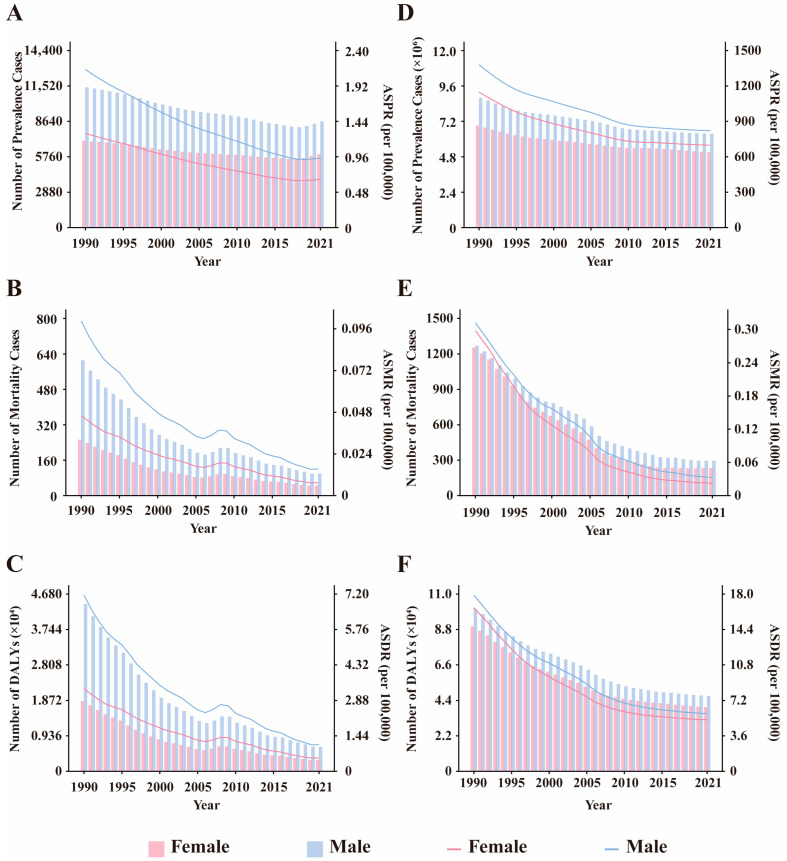
Trends in the number of prevalence, mortality, DALYs and ASPR, ASMR, ASDR by sex from 1990 to 2021. (**A**–**C**) leishmaniasis. (**D**–**F**) schistosomiasis. DALYs: disability-adjusted life years, ASPR: Age-standardized prevalence rate, ASMR: age-standardized mortality rate, ASDR: age-standardized DALYs rate.

**Figure 2:**
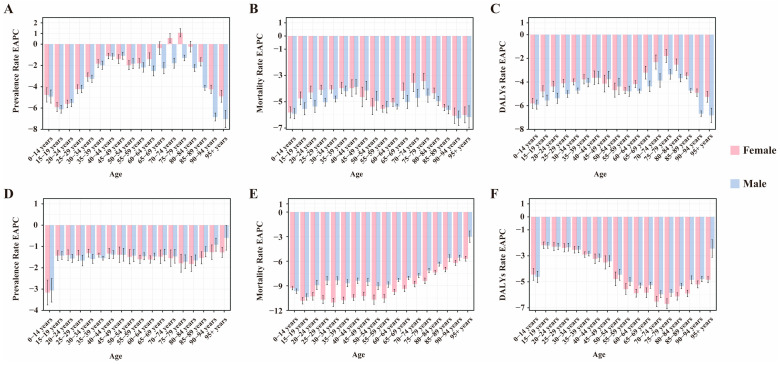
EAPC of the rate of prevalence, mortality, DALYs by sex and age from 1990 to 2021. (**A**–**C**) leishmaniasis. (**D**–**F**) schistosomiasis. EAPC: estimated annual percentage change, DALYs: disability-adjusted life years.

**Figure 3:**
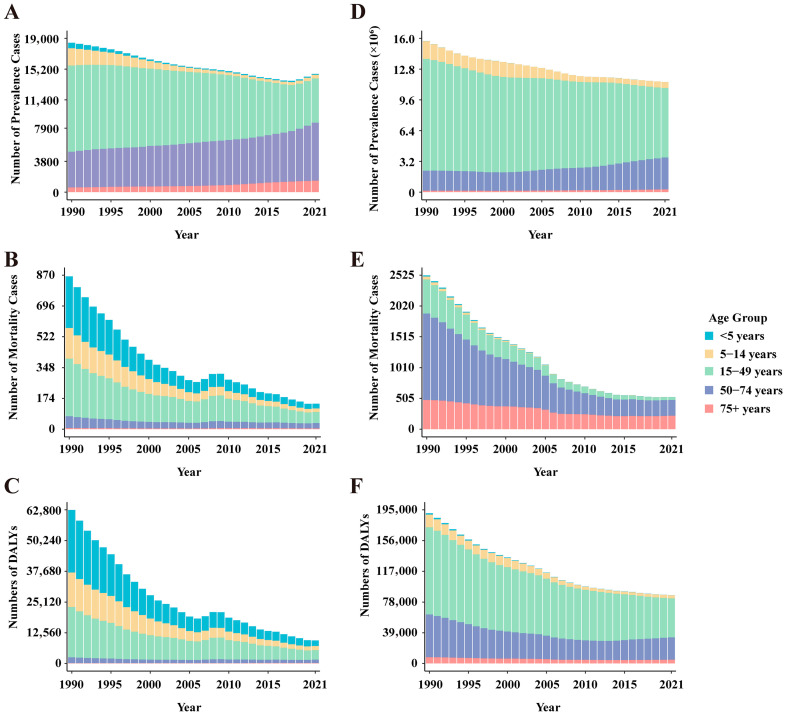
Age distribution and trends in the number of prevalence, mortality, and DALYs from 1990 to 2021. (**A**–**C**) leishmaniasis. (**D**–**F**) schistosomiasis. DALYs: disability-adjusted life years.

**Figure 4:**
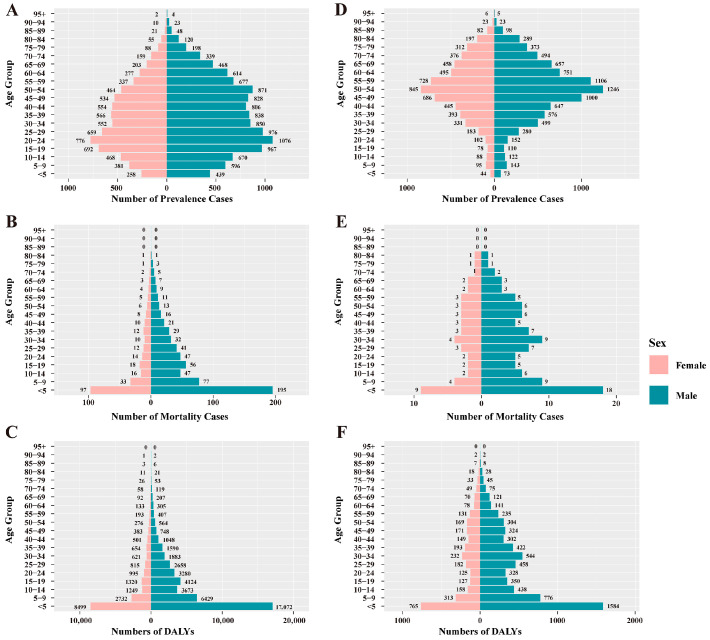
Age-sex distribution of leishmaniasis prevalence cases, mortality cases, and DALYs in 1990 (**A**–**C**) and 2021 (**D**–**F**). DALYs: disability-adjusted life years.

**Figure 5:**
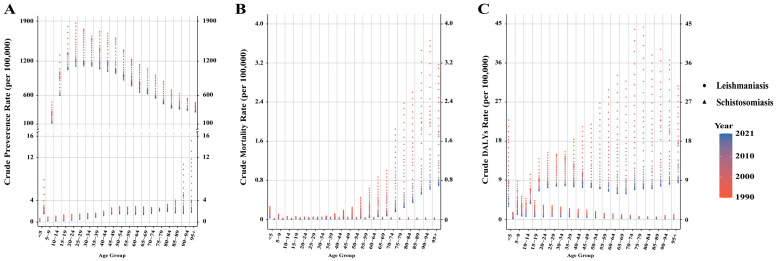
Crude prevalence (**A**), mortality (**B**), and DALY (**C**) rates of leishmaniasis and schistosomiasis in different age groups from 1990 to 2021. DALY: disability-adjusted life year.

**Figure 6:**
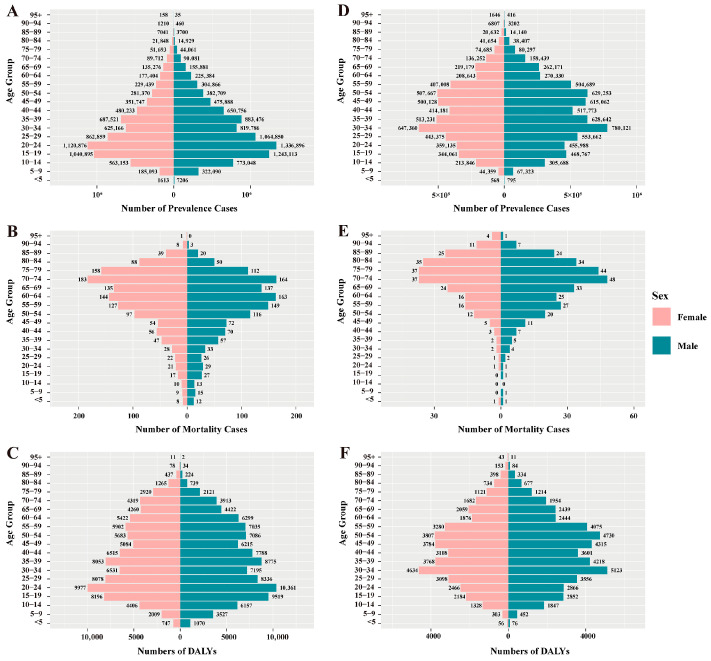
Age-sex distribution of schistosomiasis prevalence cases, mortality cases, and DALYs in 1990 (**A**–**C**) and 2021 (**D**–**F**). DALYs: disability-adjusted life years.

Overall, the prevalence of leishmaniasis and schistosomiasis in China showed a declining trend from 1990 to 2021. Among them, the number of prevalent cases of leishmaniasis decreased from 18,463 in 1990 to 14,614 in 2021, a decrease of 20.9%. Its ASPR decreased from 1.680 per 100,000 population in 1990 to 0.797 per 100,000 in 2021, with an EAPC of −2.634 (95% CI: −2.721, −2.548) (Table S1). In contrast, the prevalence burden of schistosomiasis was more substantial. During the same period, its number of prevalent cases decreased from 15,713,519 to 11,459,581, a decrease of 27.1%; its ASPR decreased from 1266.478 per 100,000 to 761.319 per 100,000, with an EAPC of −1.616 (95% CI: −1.758, −1.473) (Table S1). In terms of gender distribution, from 1990 to 2021, both the number of cases and the prevalence rate of leishmaniasis were consistently higher in men than in women. In 2021, the number of men’s cases (11,407) was approximately 1.6 times that of women (7056). In addition, the prevalence rate decreased in both sexes, with an EAPC of −2.846 (95% CI: −2.933, −2.758) in men and −2.408 (95% CI: −2.498, −2.318) in women (Table S1, Figure 1). Schistosomiasis also exhibited a pattern where both the number of cases and the prevalence rate were slightly higher in men than in women. In 2021, the number of men’s cases was approximately 6.355 million, compared to about 5.104 million in women. Similarly, the prevalence rate decreased in both sexes, with an EAPC of −1.651 (95% CI: −1.784, −1.517) in men and −1.569 (95% CI: −1.722, −1.416) in women (Table S1, Figure 1). Notably, the prevalence rate of leishmaniasis increased among women in the 70–74 and 75–79 age groups (Figure 2). Regarding age distribution, prevalent cases of leishmaniasis were primarily concentrated in the working-age population, with the fewest cases observed in the under-5 age group (Figure 3). Further analysis revealed a notable shift in the age distribution within the working-age population: the peak prevalence moved from the 20–24 age group in 1990 to the 50–54 age group in 2021 (Figure 4). Its prevalence rate was highest in the 95+ age group (Figure 5). For schistosomiasis, prevalence rates declined across all age groups, with the largest decrease observed in the 0–14 age group (Figure 2). The distribution remained concentrated in adults, but the proportion of cases in age groups > 40 years increased significantly in 2021 compared to 1990 (Figure 3 and Figure 6). The pattern of prevalence by age showed an initial increase followed by a decrease, with the highest occurring in the 20–24 age group (Figure 5).

### 3.2. The Mortality Burden of Leishmaniasis and Schistosomiasis in China

In addition to the decline in prevalence, the mortality burden of these diseases also showed a significant reduction from 1990 to 2021. Among these, the number of deaths from leishmaniasis declined overall, but experienced a brief increase between 2006 and 2009, resuming its decline after 2009 (Figure 3). Its ASMR decreased from 0.074 to 0.012, with an EAPC of −5.45 (95% CI: −5.75, −5.15) (Table S2). The number of deaths from schistosomiasis declined steadily, with ASMR falling from 0.305 to 0.027 and an EAPC of −8.16 (95% CI: −8.44, −7.88) (Figure 3, Table S2). When considering gender distribution, both the number of deaths and ASMR for leishmaniasis and schistosomiasis were higher in men than in women (Figure 1). For leishmaniasis, the mortality decreased in both sexes, with an EAPC of −5.50 (95% CI: −5.80, −5.21) in men and −5.36 (95% CI: −5.66, −5.05) in women. For schistosomiasis, the EAPC was −7.55 (95% CI: −7.79, −7.31) in men and −8.81 (95% CI: −9.14, −8.47) in women (Table S2). As for age distribution, mortality rates for both leishmaniasis and schistosomiasis decreased across all age groups (Figure 2). Deaths from leishmaniasis were predominantly concentrated in the <5 years age group, with the fewest deaths occurring among those aged 75 years and above (Figure 4). However, its mortality rate was highest in the under-5 age group (Figure 5). In contrast, deaths from schistosomiasis were concentrated in the 50–74 years age group (Figure 3 and Figure 6). And the mortality rate increased with age, reached the highest level in the 90–94 years age group (Figure 5).

### 3.3. The DALYs Burden of Leishmaniasis and Schistosomiasis in China

During the period 1990–2021, the overall burden of DALYs for PDIHs in China showed a declining trend, with leishmaniasis exhibiting a greater decrease than schistosomiasis (Table S3). The trend in the number of DALYs for leishmaniasis aligned with its mortality trend, showing fluctuations between 2006 and 2009 before resuming its decline after 2009 (Figure 3). Its ASDR decreased from 5.328 to 0.824, with an EAPC of −5.519 (95% CI: −5.797, −5.241) (Table S2). The number of DALYs for schistosomiasis also declined steadily; its ASDR decreased from 17.262 to 5.547, with an EAPC of −3.759 (95% CI: −4.032, −3.485) (Figure 3, Table S3). When stratified by sex, consistent with the mortality burden pattern, the DALY burden for PDIHs was consistently higher in men than in women (Figure 1). For leishmaniasis, the DALYs decreased in both sexes, with an EAPC of −5.575 (95% CI: −5.851, −5.298) in men and −5.427 (95% CI: −5.715, −5.137) in women. For schistosomiasis, the EAPC was −3.696 (95% CI: −3.939, −3.453) in men and −3.820 (95% CI: −4.129, −3.510) in women. (Table S3). From the perspective of age distribution, the DALY rates for both leishmaniasis and schistosomiasis decreased across all age groups (Figure 2). The number of DALYs for leishmaniasis was primarily concentrated in the <5 years age group (Figure 3 and Figure 4). For schistosomiasis, was primarily concentrated in the working-age population, with the highest appeared in younger adults (Figure 3 and Figure 6). In terms of how the DALY rate changed with age, the two diseases exhibited opposite patterns: The DALY rate for leishmaniasis decreased with increasing age, being highest among children under 5 years old. Conversely, although with transient fluctuations, the DALY rate for schistosomiasis generally increased with age (Figure 5).

### 3.4. Prediction of the Burden of Leishmaniasis and Schistosomiasis in China Using the ARIMA Model

**Figure 7:**
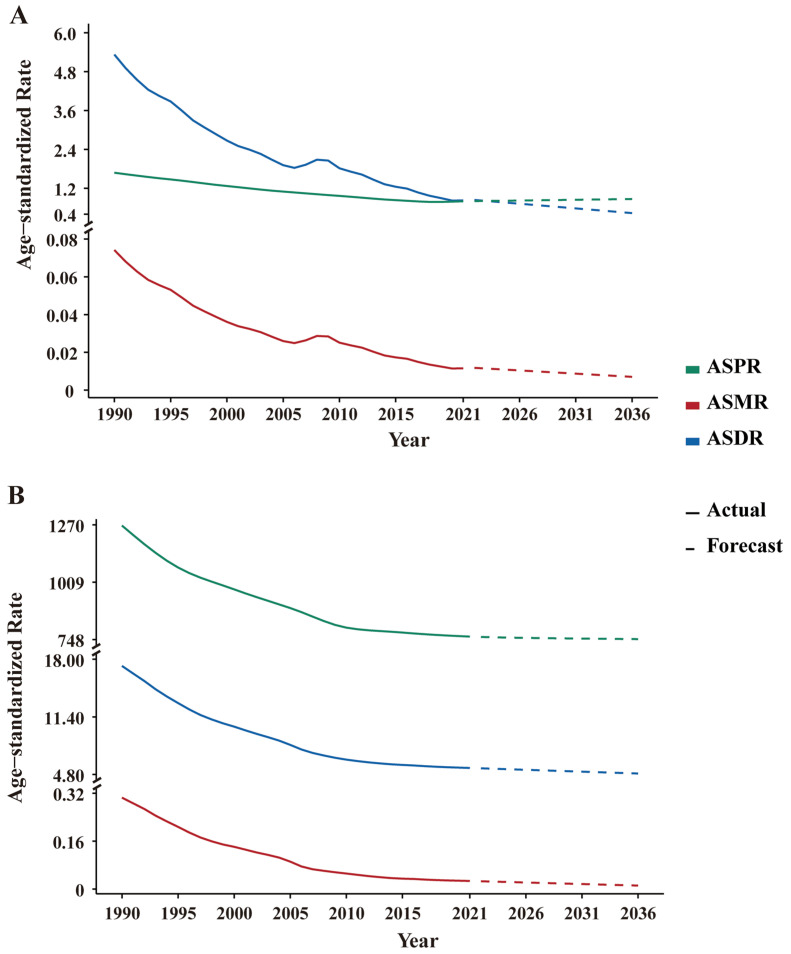
Predictions of the ASPR, ASMR and ASDR for leishmaniasis and schistosomiasis from 2022 to 2036 via the ARIMA model. (**A**) leishmaniasis. (**B**) schistosomiasis. ASPR: Age-standardized prevalence rate, ASMR: age-standardized mortality rate, ASDR: age-standardized DALYs rate, ARIMA: autoregressive integrated moving average.

The ARIMA model was adopted to forecast the disease burden of leishmaniasis and schistosomiasis in China from 2022 to 2036. The optimal model parameters and their corresponding AIC, BIC, and Ljung–Box test *p*-values are presented in Table S4. The rolling forecast validation results are presented in Tables S5 and S6. The Ljung–Box test confirms that the residuals of all models are white noise, indicating that the models are stable and demonstrate a good fit. As shown in Figure 7, it presents the time series of the age-standardized rates for leishmaniasis and schistosomiasis in China from 1990 to 2021 and the projected trend from 2022 to 2036. The prediction results indicate that the disease burden of schistosomiasis will continue to decline with minor fluctuations during 2022–2036. It is noteworthy that while the ASMR and ASDR for leishmaniasis show a decreasing trend, its ASPR is projected to experience a slight increase.

## 4. Discussion

Based on GBD 2021 data, this study reveals a significant decline in the burden of leishmaniasis and schistosomiasis in China from 1990 to 2021, with projections indicating a continued gradual decline through 2036. This success is largely attributable to sustained, multifaceted control strategies, including patriotic health campaigns, vector and intermediate host control, and improved healthcare services [8,24,33,34,35,36]. The application of emerging technologies has further enhanced the precision of vector management [24,33,37,38,39].

Despite this progress, China’s control efforts against leishmaniasis and schistosomiasis face multiple challenges. The primary challenge stems from the significant impact of climate change on disease distribution patterns: rising temperatures and altered precipitation patterns have caused vectors and intermediate hosts (e.g., *Oncomelania hupensis* snails, sandflies) to migrate towards higher latitudes and altitudes in the north, with extended activity periods. For instance, rising temperatures have expanded suitable habitats for snails and sandflies into previously non-endemic northern provinces, increasing transmission risk in new regions [18,40]. Concurrently, extreme weather events such as floods and heatwaves promote the abnormal dispersal of vectors and intermediate hosts, expanding their breeding ranges and further increasing the transmission risk of these diseases [8,18,40,41]. Secondly, although indigenous cases of these diseases are largely under control, migrant workers and international travelers returning from high-endemic regions constitute the main source of imported cases [24,42,43,44]. Therefore, China’s control strategy for these diseases requires a multi-dimensional approach: on one hand, it is essential to strengthen the construction of dynamic surveillance networks, integrate meteorological and ecological data, closely track the northward and high-altitude expansion trends of vectors and intermediate hosts like snails and sandflies, and reinforce environmental management and emergency disinfection measures in newly suitable areas. On the other hand, rapid entry screening, health tracking, and healthcare facility alert mechanisms should be implemented for returnees and travelers from high-risk regions to prevent imported cases from initiating local transmission chains.

According to GBD 2021 data, men bear a higher burden of leishmaniasis and schistosomiasis than women. This sex disparity is driven by two main factors. Firstly, occupational exposure risk differences: men typically participate more than women in outdoor occupations like agriculture, forestry, and fishing, leading to more frequent contact with parasitic vectors or contaminated water [45]. Secondly, biological immune mechanism differences: this is particularly significant in leishmaniasis [46]. Higher testosterone levels in men can directly promote parasite proliferation, inhibit macrophage bactericidal function, and induce elevated anti-inflammatory factors, weakening immune control [47]. In contrast, estrogen in women promotes Th1-type immune responses and improves parasite clearance efficiency. Additionally, incomplete X-chromosome gene inactivation in women can enhance anti-pathogen immunity, while the expression of certain autosomal genes in men correlates positively with parasite load [48]. Regarding age distribution, the study results show that the burden of leishmaniasis is concentrated in the working-age population. This is primarily due to the frequent outdoor activities of this age group and their migration, exposing them to new infection risks. China has experienced the largest annual internal population migration in the world. Millions of workers in this age group flow back and forth between rural areas and urban or industrial centers for employment, which significantly increases their exposure to different biological vectors and pathogens [49,50]. Furthermore, studies have shown that HIV-infected individuals who contract *Leishmania* parasites face a significantly higher risk of progressing to clinical visceral leishmaniasis. The disease develops more rapidly and severely in these patients, with markedly increased rates of treatment failure and relapse under standard therapy, as well as a substantially elevated mortality rate [51,52]. According to China’s HIV surveillance data, the 20–50 age group accounts for 68.0% to 77.0% of all HIV infections nationwide, with the 20–30 age group representing the highest incidence period [53]. This demographic profile highly overlaps with the age distribution of leishmaniasis cases in endemic areas, suggesting that this age bracket constitutes a high-risk population for HIV-Leishmania co-infection in these endemic regions. This convergence in age distribution has critical implications for disease prevention and control strategies. On one hand, it indicates the presence of a population more susceptible to co-infection in leishmaniasis-endemic areas. On the other hand, co-infection in this population often presents with atypical clinical manifestations, which significantly increases the difficulty of early diagnosis and clinical treatment. Additionally, against the backdrop of global aging, the disease burden of parasitic diseases is undergoing significant changes. Our analysis indicates that between 1990 and 2021, the burden of leishmaniasis and schistosomiasis has progressively shifted towards middle-aged and elderly populations. This trend highlights that the accelerating process of population aging imposes more complex demands on the long-term prevention and control of these two parasitic diseases. With advancing age, immunosenescence not only increases the susceptibility of older adults to parasitic infections but also prolongs the course of the disease and makes pathogens more difficult to eliminate following infection. Moreover, the persistence of chronic infections in the elderly population may create selective pressures that drive the evolution of parasites toward higher virulence, thereby potentially influencing transmission dynamics. Consequently, future prevention and control strategies in China should be integrated with specific diagnostic and therapeutic approaches tailored to the elderly population [54]. Projections from the ARIMA model suggest that from 2022 to 2036, the burden of schistosomiasis will continue its gradual decline. For leishmaniasis, while mortality and DALY rates are expected to decrease, a slight increase in prevalence is predicted. Given these projections, it remains imperative to sustainably strengthen surveillance of vector/intermediate host ecology and disease transmission dynamics, guarding against a potential resurgence due to relaxed monitoring, ongoing ecological changes, or increased risks of imported cases.

The GBD 2021 database provides a high-quality data foundation for the assessment of global disease burden. However, several limitations remain in this study. First, the estimates in the GBD database rely on available data sources and inherently carry uncertainty when actual disease burden data are missing [10,55]. Furthermore, because GBD data relies on standardized modeling and cross-country data integration, its globally uniform models struggle to fully capture dynamic changes such as locally specific epidemiological characteristics and rapid policy effects, which may lead to discrepancies with real-world data [56,57]. Additionally, the ARIMA model itself has certain limitations: it requires a substantial amount of data and primarily models the intrinsic dynamics of the time series, making it difficult to directly incorporate the influence of external drivers such as climate change and adjustments in control policies on these diseases [55,58].

## 5. Conclusions

This study demonstrates a significant and sustained decline in the overall burden of leishmaniasis and schistosomiasis in China from 1990 to 2021. However, our analysis highlights persistent challenges. Critically, while ARIMA projections suggest a continued decline for schistosomiasis through 2036, leishmaniasis prevalence may see a slight increase, signaling a need for focused intervention. These gains are threatened by climate-change-driven habitat expansion of vectors and intermediate hosts, alongside the ongoing risk of imported cases from endemic regions. Therefore, consolidating past achievements and addressing future threats necessitates a dynamic and integrated strategy. This includes strengthening eco-epidemiological surveillance networks, rigorously managing imported cases, implementing targeted interventions for high-risk populations, and fostering multi-sectoral collaboration. Such a comprehensive approach is essential to mitigate the potential rise in leishmaniasis and to ultimately achieve the goal of eliminating both diseases.

## Data Availability

The data supporting the findings of this study were sourced from the GBD 2021 database, which can be freely accessed at http://ghdx.healthdata.org/gbd-results-tool (accessed on 10 June 2025).

## Supplementary Materials

The following supporting information can be downloaded at https://www.mdpi.com/article/10.3390/pathogens15030340/s1: Table S1: Prevalence of leishmaniasis and schistosomiasis in China in 1990 and 2021, and the temporal trends from 1990 to 2021; Table S2: Mortality of leishmaniasis and schistosomiasis in China in 1990 and 2021, and the temporal trends from 1990 to 2021; Table S3: DALYs of leishmaniasis and schistosomiasis in China in 1990 and 2021, and the temporal trends from 1990 to 2021; Table S4: ARIMR model parameters and their corresponding AIC, BIC and Ljung–Box test *p* value; Table S5: The rolling forecast validation results of leishmaniasis; Table S6: The rolling forecast validation results of schistosomiasis.

## Abbreviations

The following abbreviations are used in this manuscript:

PDIHs	Diseases caused by parasitic with invertebrate hosts
GBD	Global Burden of Diseases
DALYs	Disability-adjusted life years
ARIMA	Autoregressive integrated moving average
EAPC	Estimated annual percentage change
WHO	World Health Organization
ASR	Age-standardized rate
ASPR	Age-standardized prevalence rate
ASMR	Age-standardized mortality rate
ASDR	Age-standardized DALYs rate
CI	Confidence interval
