# Supplementary material for: Diseases Caused by Parasites with Invertebrate Hosts in China: Burden and Trends of Leishmaniasis and Schistosomiasis
Source: Pathogens. 2026 Mar 23;15(3):340. doi: 10.3390/pathogens15030340 (PMC13028703; doi:10.3390/pathogens15030340)
Supplement: Supplementary file 1 [file pathogens-15-00340-s001.zip › S2 Table.pdf]

Table S2. Mortality of leishmaniasis and schistosomiasis in China in 1990 and 2021, and the temporal trends from 1990 to 2021

Characteristics		Number (95% UI)		Percentage Change (%) (95% UI)	ASMR per 100,000 (95% UI)		EAPC (95% UI)
		1990	2021	1990 to 2021	1990	2021	1990 to 2021
Leishmaniasis	Sex						
	Both	863.686 (0.109, 4752.115)	144.145 (0.020, 759.021)	-83.310 (-85.842, -74.588)	0.074 (0.000, 0.400)	0.012 (0.000, 0.064)	-5.450 (-5.746, -5.154)
	Males	611.202 (0.073, 3387.642)	99.242 (0.012, 528.673)	-83.763 (-86.560, -74.493)	0.100 (0.000, 0.546)	0.015 (0.000, 0.086)	-5.504 (-5.798, -5.209)
	Females	252.485	44.902	-82.216	0.046	0.007	-5.356

		(0.036, 1327.608)	(0.008, 220.766)	(-85.584, -71.282)	(0.000, 0.238)	(0.000, 0.039)	(-5.664, -5.047)
	Sex						
Schistosomiasis	Both	2522.896 (2148.639, 2928.568)	527.289 (423.269, 638.599)	-79.100 (-83.806, -72.819)	0.305 (0.260, 0.353)	0.027 (0.022, 0.033)	-8.163 (-8.442, -7.884)
	Males	1269.492 (1014.721, 1541.136)	294.298 (227.219, 379.279)	-76.818 (-84.047, -67.036)	0.312 (0.252, 0.374)	0.033 (0.025, 0.042)	-7.548 (-7.788, -7.307)
	Females	1253.404 (987.007, 1530.206)	232.991 (179.354, 304.520)	-81.411 (-86.356, -73.522)	0.298 (0.236, 0.363)	0.022 (0.017, 0.029)	-8.807 (-9.139, -8.473)
