# Supplementary material for: Diseases Caused by Parasites with Invertebrate Hosts in China: Burden and Trends of Leishmaniasis and Schistosomiasis
Source: Pathogens. 2026 Mar 23;15(3):340. doi: 10.3390/pathogens15030340 (PMC13028703; doi:10.3390/pathogens15030340)
Supplement: Supplementary file 1 [file pathogens-15-00340-s001.zip › S3 Table.pdf]

Table S3. DALYs of leishmaniasis and schistosomiasis in China in 1990 and 2021, and the temporal trends from 1990 to 2021

Characteristics		Number (95% UI)		Percentage Change (%) (95% UI)	ASDR per 100,000 (95% UI)		EAPC (95% UI)
		1990	2021	1990 to 2021	1990	2021	1990 to 2021
Leishmaniasis	Sex						
	Both	62751.444 (661.299, 347023.494)	9456.490 (504.387, 48377.096)	-84.930 (-87.721, -9.708)	5.328 (0.059, 29.098)	0.824 (0.030, 4.500)	-5.519 (-5.797, -5.241)
	Males	44189.590 (429.980, 246686.547)	6484.150 (310.613, 34340.392)	-85.327 (-88.066, -14.229)	7.157 (0.076, 39.658)	1.079 (0.036, 6.063)	-5.575 (-5.851, -5.298)
	Females	18561.854	2972.340	-83.987	3.352	0.539	-5.427

		(224.542, 98610.783)	(189.444, 14214.220)	(-87.489, 1.680)	(0.040, 17.610)	(0.024, 2.787)	(-5.715, -5.137)
	Sex						
Schistosomiasis	Both	190711.522 (130309.400, 300095.210)	86748.829 (43666.062, 167202.370)	-54.513 (-66.913, -43.643)	17.262 (12.126, 26.173)	5.547 (2.648, 11.025)	-3.759 (-4.032, -3.485)
	Males	100820.184 (66717.926, 163231.856)	46867.934 (23788.928, 90031.044)	-53.513 (-66.263, -43.540)	17.877 (12.367, 27.631)	5.863 (2.837, 11.378)	-3.696 (-3.939, -3.453)
	Females	89891.339 (61631.325, 140449.606)	39880.895 (20007.895, 77106.349)	-55.634 (-68.406, -43.643)	16.597 (11.717, 25.001)	5.229 (2.482, 10.581)	-3.820 (-4.129, -3.510)
