# Supplementary material for: Diseases Caused by Parasites with Invertebrate Hosts in China: Burden and Trends of Leishmaniasis and Schistosomiasis
Source: Pathogens. 2026 Mar 23;15(3):340. doi: 10.3390/pathogens15030340 (PMC13028703; doi:10.3390/pathogens15030340)
Supplement: Supplementary file 1 [file pathogens-15-00340-s001.zip › S4 Table.pdf]

Table S4. ARIMR model parameters and their corresponding AIC, BIC and Ljung–Box test *p* value

Diseases	Measures	Parameters	AIC	BIC	Ljung–Box test p value
**Leishmaniasis**	ASPR	ARIMA(3,1,3)	-257.06	-247.02	0.0763
	ASMR	ARIMA(3,1,0)	-331.15	-325.42	0.9014
	ASDR	ARIMA(0,2,2)	-68.27	-64.06	0.6443
**Schistosomiasis**	ASPR	ARIMA(1,2,1)	88.07	92.28	0.1279
	ASMR	ARIMA(0,2,1)	-280.50	-277.70	0.8273
	ASDR	ARIMA(0,2,1)	-85.92	-83.11	0.8635
