# Supplementary material for: Diseases Caused by Parasites with Invertebrate Hosts in China: Burden and Trends of Leishmaniasis and Schistosomiasis
Source: Pathogens. 2026 Mar 23;15(3):340. doi: 10.3390/pathogens15030340 (PMC13028703; doi:10.3390/pathogens15030340)
Supplement: Supplementary file 1 [file pathogens-15-00340-s001.zip › S5 Table.pdf]

Table S5. The rolling forecast validation results of leishmaniasis

	Year	Actual	Forecast	MAPE
ASPR	2007	1.043156438	1.042725518	0.35%
	2008	1.015838632	1.016159705	
	2009	0.988966486	0.988520826	
	2010	0.964477003	0.962094340	
	2011	0.937516798	0.939987519	
	2012	0.908955124	0.910556593	
	2013	0.880189465	0.880393450	
	2014	0.853594813	0.851423807	
	2015	0.833034287	0.827000161	
	2016	0.812897549	0.812473760	
	2017	0.792645993	0.792066254	
	2018	0.778319819	0.772606321	
	2019	0.777333419	0.763993645	
	2020	0.786579598	0.785489794	
	2021	0.796591232	0.802266336	
ASMR	2007	0.026347796	0.024590816	5.24%
	2008	0.028673109	0.029068900	
	2009	0.028422150	0.030667780	
	2010	0.025131750	0.026288323	
	2011	0.023718642	0.020945282	
	2012	0.022487961	0.025808174	
	2013	0.020354471	0.020109632	
	2014	0.018354967	0.018320340	
	2015	0.017333939	0.015898690	
	2016	0.016607047	0.016769495	
	2017	0.014917080	0.015914641	
	2018	0.013526162	0.013265577	
	2019	0.012494758	0.012342703	
	2020	0.011450334	0.011364006	
	2021	0.011533396	0.010340120	
ASDR	2007	1.926131627	1.801850063	4.54%
	2008	2.082255790	2.113101067	
	2009	2.055207926	2.180499260	
	2010	1.817081900	1.892242137	
	2011	1.713599074	1.574063958	
	2012	1.622147931	1.875830178	
	2013	1.467367956	1.449294646	
	2014	1.325015372	1.319862240	
	2015	1.246356488	1.157768686	
	2016	1.188976992	1.197429444	

2017	1.067973860	1.093274234
2018	0.969374698	0.949007886
2019	0.897344617	0.884875755
2020	0.820637362	0.817047487
2021	0.823509931	0.737736160
