# Supplementary material for: Diseases Caused by Parasites with Invertebrate Hosts in China: Burden and Trends of Leishmaniasis and Schistosomiasis
Source: Pathogens. 2026 Mar 23;15(3):340. doi: 10.3390/pathogens15030340 (PMC13028703; doi:10.3390/pathogens15030340)
Supplement: Supplementary file 1 [file pathogens-15-00340-s001.zip › S6 Table.pdf]

Table S6. The rolling forecast validation results of schistosomiasis

	Year	Actual	Forecast	MAPE
ASPR	2007	852.025387993	851.261483991	0.10%
	2008	831.916518598	829.839999777	
	2009	814.341860377	813.488746273	
	2010	801.981970053	799.950586359	
	2011	794.622600569	795.715421491	
	2012	789.808997025	790.993726484	
	2013	786.456880755	785.135039272	
	2014	783.309299946	783.543036788	
	2015	779.639148071	779.518712901	
	2016	775.601358390	775.108631080	
	2017	772.012772800	771.380510507	
	2018	768.871487410	769.283546176	
	2019	766.030604205	766.069731109	
	2020	763.552929712	764.025117606	
	2021	761.318671500	760.947221107	
ASMR	2007	0.066118187	0.059303105	2.38%
	2008	0.060890191	0.056789864	
	2009	0.056080834	0.055662196	
	2010	0.051842037	0.051271477	
	2011	0.047424316	0.047848221	
	2012	0.043160108	0.042795300	
	2013	0.039676234	0.039078717	
	2014	0.036574567	0.036493264	
	2015	0.034780065	0.033513807	
	2016	0.033705659	0.033628298	
	2017	0.031648908	0.032669030	
	2018	0.030042138	0.029066626	
	2019	0.028802014	0.028932179	
	2020	0.027944321	0.027495762	
	2021	0.027048680	0.027313546	
ASDR	2007	7.269966664	7.137679766	0.46%
	2008	6.977412740	6.872888745	
	2009	6.715208951	6.705033285	
	2010	6.504157624	6.457807287	
	2011	6.333217850	6.315264878	
	2012	6.188542305	6.170875298	
	2013	6.070097922	6.052489352	
	2014	5.966815266	5.960281480	
	2015	5.895134239	5.866750793	
	2016	5.833703501	5.837496910	

2017	5.751942822	5.770397214
2018	5.686319667	5.661016399
2019	5.630859133	5.633256689
2020	5.587849022	5.574207219
2021	5.547372932	5.551580455
