# Supplementary material for: Diseases Caused by Parasites with Invertebrate Hosts in China: Burden and Trends of Leishmaniasis and Schistosomiasis
Source: Pathogens. 2026 Mar 23;15(3):340. doi: 10.3390/pathogens15030340 (PMC13028703; doi:10.3390/pathogens15030340)
Supplement: Supplementary file 1 [file pathogens-15-00340-s001.zip › S1 Table.pdf]

Table S1. Prevalence of leishmaniasis and schistosomiasis in China in 1990 and 2021, and the temporal trends from 1990 to 2021

Characteristics		Number (95% UI)		Percentage Change (%) (95% UI)	ASPR per 100,000 (95% UI)		EAPC (95% UI)
		1990	2021	1990 to 2021	1990	2021	1990 to 2021
Leishmaniasis	Sex						
	Both	18463.089 (5251.582, 53150.583)	14613.770 (6123.130, 35206.675)	-20.849 (-34.000, 24.296)	1.680 (0.478, 4.851)	0.797 (0.372, 1.806)	-2.634 (-2.721, -2.548)
	Males	11407.401 (3271.938, 32587.406)	8646.169 (3703.958, 20987.935)	-24.206 (-36.896, 19.473)	2.144 (0.608, 6.162)	0.942 (0.439, 2.170)	-2.846 (-2.933, -2.758)
	Females	7055.688	5967.601	-15.421	1.278	0.655	-2.408

		(1928.237, 21435.308)	(2428.660, 14990.894)	(-28.380, 34.405)	(0.349, 3.883)	(0.300, 1.546)	(-2.498, -2.318)
	Sex						
Schistosomiasis	Both	15713519.290 (12373489.034, 21733892.941)	11459581.102 (8953886.987, 15054894.915)	-27.072 (-35.424, -18.542)	1266.478 (1007.530, 1754.970)	761.319 (579.993, 991.058)	-1.616 (-1.758, -1.473)
	Males	8799213.338 (6959226.257, 12246939.551)	6355165.181 (4999292.886, 8487841.220)	-27.776 (-35.987, -19.319)	1379.244 (1097.812, 1906.351)	820.792 (630.469, 1077.772)	-1.651 (-1.784, -1.517)
	Females	6914305.952 (5464999.105, 9477879.214)	5104415.921 (3934410.179, 6799886.018)	-26.176 (-34.816, -16.722)	1146.103 (916.722, 1577.198)	697.956 (525.223, 914.586)	-1.569 (-1.722, -1.416)
